# Coumarin-Containing Polymers for High Density Non-Linear Optical Data Storage

**DOI:** 10.3390/molecules21020147

**Published:** 2016-01-26

**Authors:** Denis Gindre, Konstantinos Iliopoulos, Oksana Krupka, Marie Evrard, Emilie Champigny, Marc Sallé

**Affiliations:** 1 Laboratory MOLTECH-Anjou, CNRS UMR 6200, University of Angers, 2 Bd Lavoisier, 49045 Angers CEDEX, France; kostiliopoulos@gmail.com (K.I.); marie.evrard@univ-angers.fr (M.E.); emilie.champigny@gmail.com (E.C.); marc.salle@univ-angers.fr (M.S.); 2 Department of Chemistry, Taras Shevchenko National University of Kyiv, 60 Volodymyrska, 01033 Kyiv, Ukraine; oksana_krupka@yahoo.com

**Keywords:** optical data storage, coumarin, two-photon absorption, second harmonic generation

## Abstract

Optical data storage was performed with various thin polymer films containing coumarin-based derivatives and by using femtosecond laser pulses as well as two-photon absorption processes. Exploring the photodimerization attribute of coumarin derivatives and using appropriate irradiation wavelengths, recording/erasing processes could be carried out in the same area. Second harmonic generation microscopy was used to read the stored information.

## 1. Introduction

A key challenge of the 21st century concerns the management of information. The exponential increase of the amount of digital information requires continuous improvement of storage capacities. The last few years have seen intensive research focusing on novel materials and/or new experimental techniques suitable for optical data storage (ODS) as a complementary approach to magnetic hard drives and high capacities flash memories. New materials, based for instance on DNA [1] or nanostructured glass [2], have been used for ODS and there has been concurrent progress made for advanced techniques [3]. Despite these ongoing advances, the market demand for light and inexpensive high capacity media remains strong and organic materials based on polymers functionalized with active chromophores constitute a promising approach. In particular, the possibility to stimulate photochromic materials in order to modulate their linear or nonlinear optical properties constitutes an important breakthrough regarding applications. For those related to ODS, two-photon processes are often used as they provide high storage capacity, due to the quadratic dependence on the incident light intensity. The main advantage of two-photon absorption-induced photochemistry lies in the precise three dimensional (3D) spatial control of the photoreactions. The use of two-photon processes properties to improve data storage was introduced by the Rentzepis group [4,5] and several works have been reported since, including rewritable ODS based on spironaphthoxazine [6], two-photon optical storage by fluorescence modulation [7,8], as well as 3D ODS using photochromic materials [9]. Another important light-related effect is second-harmonic generation (SHG), which corresponds to a nonlinear optical process of conversion of two photons at fundamental frequency into one photon at doubled frequency. Importantly, photoinduced chemical modifications carried out at the molecular level in the bulk of the material can induce a local modification of the SHG response of the material. This detection parameter, which is inherently nonlinear and harder to measure than other linear optical contrasts, is interesting in terms of high density volumetric optical storage. On this basis, some of us reported ODS by modulation of the SHG response occurring upon the *cis*-*trans* isomerization of azo-dyes chromophores grafted in a polymeric matrix [10,11,12]. Others proposed an approach based on quantum dots [13].

**Scheme 1:**
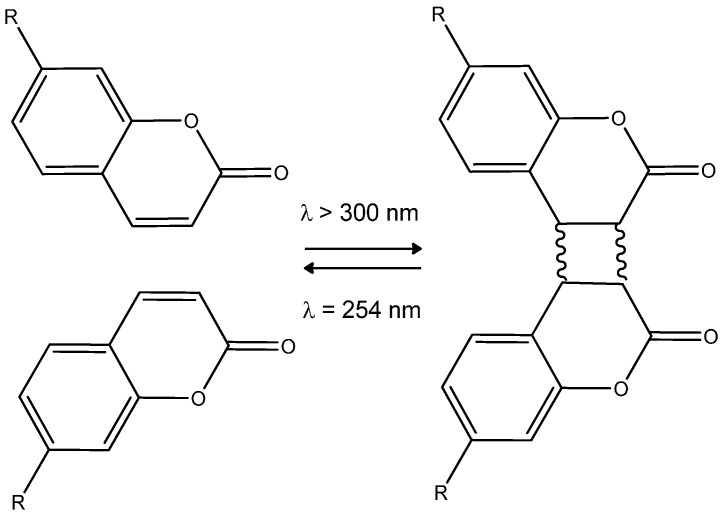
Reversible photodimerization process of coumarin (four possible isomers of the dimeric form can be obtained).

Coumarins and their analogues are well-known for their photochemical and photophysical properties [14,15,16]. In particular, some coumarin derivatives are able to undergo a photoinduced cyclodimerization [17,18] to a cyclobutane-based dimer. The reverse process (photocleavage) can be induced upon irradiation with higher energy photons (wavelength = 254 nm) [19,20,21]. We previously reported the remarkable ability of a coumarin-based copolymer to undergo a reversible writing-erasing process [22,23,24] and its potential use in particular for anti-counterfeiting applications [25]. Optical storage based on modulation of the SHG response in coumarin-based materials involves the photo-induced reaction shown in Scheme 1. A coumarin derivative cyclodimerizes to a stable cyclobutane-based dimer upon irradiation at *λ* > 300 nm (four isomeric configurations, *i.e.*, *syn* head-to-head, *anti* head-to-head, *syn* head-to-tail, and *anti* head-to-tail are possible), whereas the reverse photocleavage reaction occurs at shorter wavelengths. When a donating substituent is located on the 7-position of the coumarin unit (typically R = OR′ (alcoxy)), the corresponding chromophore presents a suitable π-delocalization profile over the entire molecule with a suitable dipole moment to get a SHG response. This is no longer the case with the dimeric form, once the cyclobutane ring is formed, the latter fragment preventing the π-delocalization. Consequently it is possible, providing a previous spatial organization of the coumarin dipoles by means of an externally applied electric field (corona poling) [26], to address locally the SHG response in the material, from a high value (monomer form) to a low value (dimer form). It has to be noted that when applied to the case of a functionalized polymer bearing pendant coumarin units, the cyclodimerization process, being intermolecular, gives rise to a cross-linking of the corresponding polymer, an issue which is well documented [14,18,27].

Even if coumarins appear more appealing than azobenzenes with respect to ODS applications because of their greater transparency in the visible range, the fact remains that their nonlinear responses are weak and especially the contrast between the response of the monomeric coumarin form and the cross-linked form remains low. The objective of this study is therefore to determine the best composition of the copolymer that affords the highest contrast of the SHG response. In this full-paper, we report an exhaustive study related to the synthesis, characterization and nonlinear optical (NLO) properties of a family of methacrylic copolymers incorporating coumarin side-groups as NLO-active fragments. A comparative study addressing the NLO response of the corresponding films as a function of their molecular formula has been carried out and allows to address the critical parameters for application in ODS.

## 2. Results and Discussion

We first developed the synthesis of a series of twenty-one methacrylic copolymers **P1**–**P21** bearing coumarin side groups.

### 2.1. Synthesis and Characterization of Copolymers ***P1**–**P21***

**Scheme 2:**
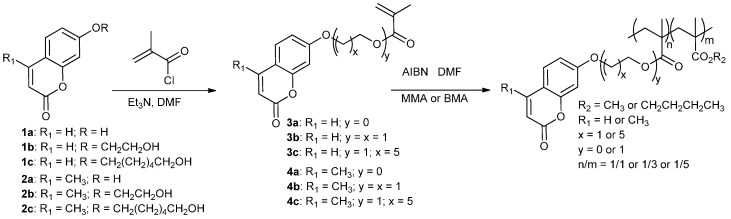
Synthesis of coumarin-functionalized methacrylic ester co-polymers (MMA: methyl methacrylate; BMA: butyl methacrylate).

The coumarin-based methacrylic monomers **3a**–**c** and **4a**–**c** were synthesized by reaction of methacryloyl chloride with the coumarin alcohol derivatives **1a**–**c** and **2a**–**c**, respectively, in the presence of triethylamine (Scheme 2). The corresponding copolymers were obtained by radical polymerization of these monomers with methyl methacrylate (MMA) or butyl methacrylate (BMA), using 2,2-azobis(isobutyronitrile) (AIBN) as radical initiator. The structures of the twenty-one copolymers were confirmed by ^1^H-NMR spectroscopy and a reasonable accord was found between the observed n/m values in the polymers ((n/m)_obs_ in Table 1 and Table 2) and the respective amounts of both monomers which were introduced ((n/m)_0_). Nevertheless, as expected from their less hindered character, the BMA or MMA motifs are generally found in slight excess in the final composition of the polymers (m values). Glass transition temperatures (T_g_) are collected in Table 1 and Table 2. As expected, a higher ratio in coumarin motif results in a higher T_g_ when MMA (R_2_ = CH_3_) is used. Also, introduction of conformationally flexible alkyl chains as linkers of the coumarin chromophore (y = 1 and x = 5) and/or in the methacrylate co-monomer (R_2_ = *n*-butyl) leads to a systematically lower value of T_g_. Finally, the copolymers’ molecular weights are in the 11700–52100 range and polydispersity indices are 1.4–2 as determined by gel permeation chromatography (Table 1 and Table 2).

**Table 1:** Results of radical polymerization of 10% methacrylic monomers (case of monomers **3a** and **4a** (y = 0)) in DMF at 80 °С (argon atmosphere, AIBN 1%).

Polymer	R_1_	R_2_	(n/m)_0_^a^	(n/m)_obs_^b^	Mn ^c^	Mw ^c^	Mv ^c^	Ipd ^c^	T_g_^d^
**P1 ^e^**	H		-	-	-	-	-	-	120
**P2**	H	CH_3_	1:1	1:1.4	14,700	28,100	25,600	1.9	112
**P3**	H	CH_3_	1:3	1:2.95	14,500	24,800	22,900	1.7	88
**P4**	H	CH_3_	1:5	1:5.57	21,900	34,900	32,500	1.6	83
**P5**	H	(CH_2_)_3_CH_3_	1:1	1:1.16	15,900	31,400	28,100	1.9	85
**P6**	H	(CH_2_)_3_CH_3_	1:3	1:3.9	27,200	52,100	47,000	1.9	73
**P7 ^e^**	CH_3_		-	-	-	-	-	-	152
**P8**	CH_3_	CH_3_	1:1	1:1.18	21,200	34,000	31,000	1.6	143
**P9**	CH_3_	CH_3_	1:3	1:3	18,900	33,000	30,400	1.7	110
**P10**	CH_3_	CH_3_	1:5	1:5.52	20,500	33,200	30,800	1.6	103
**P11**	CH_3_	(CH_2_)_3_CH_3_	1:1	1:1.3	18,600	36,000	32,800	1.9	81
**P12**	CH_3_	(CH_2_)_3_CH_3_	1:3	1:3	17,000	32,100	29,300	1.9	76

^a^ Molar ratio of monomers; ^b^ n/m stoichiometry in the copolymer as determined from ^1^H-NMR integration; ^c^ Measured by gel permeation chromatography; ^d^ Measured by differential scanning calorimetry; ^e^ No co-monomer added; the resulting polymer is not soluble in THF.

**Table 2:** Results of radical polymerization of 10% methacrylic ester monomers (case of monomers **3b**,**c** and **4b**,**c** (y = 1)) in DMF at 80 °С (argon atmosphere, AIBN 1%).

Polymer	R_1_	x	R_2_	(n/m)_0_^a^	(n/m)_obs_^b^	Mn ^c^	Mw ^c^	Mv ^c^	Ipd ^c^	T_g_^d^
**P13**	CH_3_	1	CH_3_	1:3	1:4	-	16,900	16,900	1.4	90
**P14**	CH_3_	5	CH_3_	1:1	1:1.32	13,600	25,000	23,000	1.8	40
**P15**	CH_3_	5	CH_3_	1:3	1:3.21	17,900	36,600	24,000	2	47
**P16**	CH_3_	1	CH_3_	1:1	1:1.11	6000	11,700	10,100	2	85
**P17**	CH_3_	5	CH_3_	1:5	1:6	16,500	29,700	24,700	1.8	80
**P18**	H	1	CH_3_	1:3	1:2.56	13,000	27,000	14,700	2.0	93
**P19**	H	1	CH_3_	1:1	1:1.2	9700	17,200	18,900	1.7	81
**P20**	H	5	CH_3_	1:3	1:3	16,000	30,400	-	1.9	73
**P21**	H	5	CH_3_	1:1	1:1.75	12,000	23,500	21,000	1.95	50

^a^ Molar ratio of monomers; ^b^ n/m stoichiometry in the copolymer as determined from ^1^H-NMR integration; ^c^ Measured by gel permeation chromatography; ^d^ Measured by differential scanning calorimetry.

### 2.2. UV-Vis Spectroscopy

**Figure 1:**
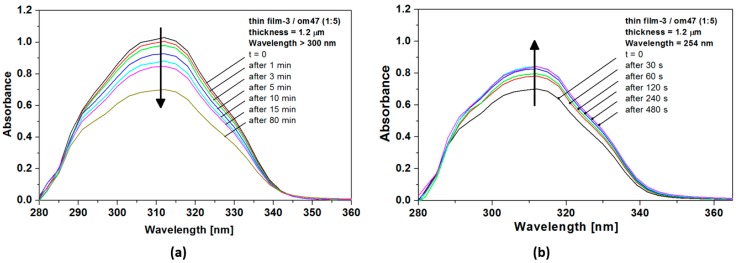
(**a**) Evolution of the absorption spectrum of a **P17** thin film (1.2 μm) with time upon irradiation at λ > 300 nm; (**b**) Changes of the absorption spectrum of cross-linked **P17** thin film upon irradiation at 254 nm.

Photoinduced dimerization and photocleavage studies were conducted on thin films of the copolymers. Their optical absorption spectra show one major absorption region, assigned to the absorption of the conjugated chromophore. During irradiation at λ > 300 nm, a decrease of the absorbance assigned to π–π* transition of the coumarin unit is observed, as illustrated for instance with the case of polymer **P17** which evolves to the corresponding cross-linked form (Figure 1a). The decreasing of the optical density is observed for all polymers studied, as expected from the light-induced dimerization reaction to the corresponding less conjugated cyclobutane dimeric form. Conversely, irradiation of the latter film at λ = 254 nm (Figure 1b) shows the progressive restoration of the π–π* signature of the coumarin backbone which corresponds to the reverse reaction, *i.e.*, photocleavage of the dimeric form. The trends observed are the same for all polymer films studied, which all exhibit both reverse photoinduced processes, depending on the irradiation wavelength (Figures S1–S6).

### 2.3. Second Order Nonlinear Response

The thin films exhibit a centrosymmetric attribute as the molecules are randomly oriented. In order to break this centrosymmetry, we used corona poling, which allows an alignment of the molecules by means of an externally applied electric field (see Experimental Section). Note that we excluded from these poling experiments all polymers presenting low T_g_ values (typically <80 °C) since it is well-established that in this case, a dipole relaxation occurs subsequently to the corona poling, a behaviour which is not compatible with SHG. We therefore discarded several polymers from the following NLO studies, in particular those bearing long alkyl linkers (e.g., **P14**, **P15**, **P17**, **P20**, **P21**) (Table 2).

Two different sequences to perform ODS were performed. The first one was based on: (i) irradiation of the spin-coated film to induce photo-dimerization, (ii) corona-poling, (iii) SHG scanning to read the data. The second sequence involves inversion of steps (i) and (ii). We found out that the latter sequence results in a very significant improvement of the ODS quality, manifested by a higher SHG contrast between irradiated and non-irradiated areas. This difference is assigned to a more efficient orientation of the molecules upon corona-poling at the coumarin monomeric state compared to the cyclobutane dimeric one, which is a result of the higher dipole moment of the π-conjugated monomer. On this basis, we decided to perform the ODS studies with samples which are corona-poled before the recording process. 

Two different experimental approaches were used for the data recording/reading. In the first case, the sample was displaced relative to the laser beam by means of a three-dimensional stage, while in the second approach the sample was kept immobile and rapidly scanned by the laser beam using an X–Y scanner. We found that the latter set-up offered a higher speed (a few microseconds for one pixel compared to a few ms with the first configuration) for both the data writing and reading [24]. For ODS applications, efficient SHG is required as well as a significant SHG contrast between the “0” and the “1” bits, which correspond to the two different molecular states. We previously determined [23] the absolute values of the second order NLO susceptibilities, before and after the dimerization process, for three coumarin-based polymers (**P8**, **P9**, **P11**). These values were obtained by the Maker fringes technique using picosecond laser pulses. However the low repetition rate of a picosecond laser system cannot be adapted for data storage purposes. We therefore used a Ti:Sapphire laser source (Tsunami, Spectra-Physics, Santa Clara, CA, USA) with 120 fs pulse duration and a repetition rate of 80 MHz for our developed ODS setup (see Experimental Section). An additional clear advantage of femtosecond laser pulses is that an intense light peak power is obtained at the focal point of an objective lens, offering the possibility to induce two-photon absorption phenomena, which in turn induces a higher localized interaction area. In this way a 3D optical data storage in the bulk of the thin films could be achieved.

### 2.4. Comparative Investigation of the Optical Data Recording Efficiency

A detailed study of the influence of the molecular structure on the recording efficiency was carried out, in order to find the most promising systems for optical storage devices. Many films of coumarin-polymers were thus prepared and corona-poled. The goal of this study was to determine the highest SHG contrast between the non-irradiated areas (corresponding to bit “0”, *i.e.*, coumarin monomers) and the laser-irradiated areas (corresponding to bit “1”, *i.e*., dimerized coumarin). Special attention was paid to keep the experimental conditions unchanged during the different experiments in order to enable direct comparison between the samples. The fixed parameters include the laser excitation wavelength (fixed at 700 nm), the microscope objective lens (SLMPLN20X, Olympus, Tokyo, Japan, numerical aperture = 0.25) and the laser power (50 mW). The bits “0” and “1” were determined by the SHG level of the non-irradiated (high SHG signal) and the irradiated zones (low SHG signal) respectively.

**Scheme 3:**
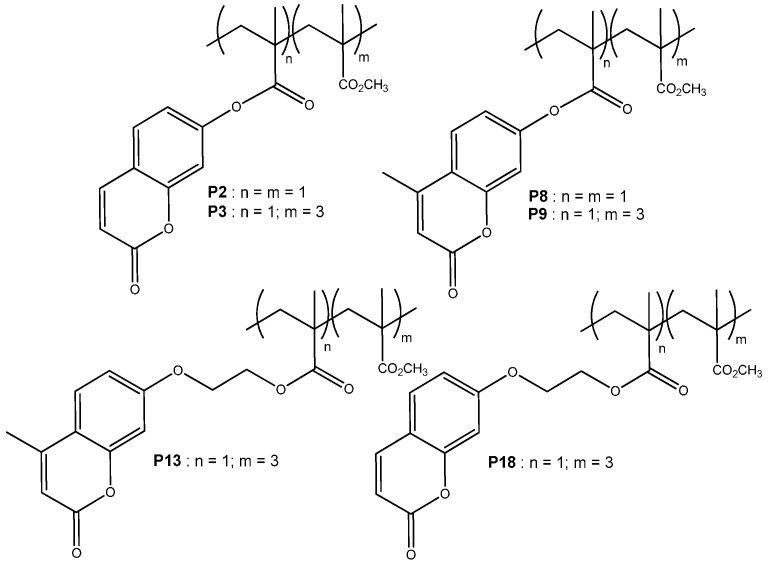
Coumarin-based copolymers investigated for optical storage applications.

The SHG contrasts for six polymers films (Scheme 3) are shown in Table 3, where the values of measured SHG signals in arbitrary units and the corresponding normalized values are reported as well as the contrast, defined by the ratio (SHG_Max_ − SHG_Min_)/(SHG_Max_ + SHG_Min_). The ideal material for ODS applications should be have a contrast of 100%.

In order to address the influence of the linker length, comparative studies were first performed between **P13** and **P9** on one hand, and between **P18** and **P3** on the other hand. These co-polymers all exhibit the same n/m (= 1/3) ratio and whereas the former pair involves a methylcoumarin derivative, the latter one is related to unsubstituted coumarin. In addition, in each pair one member corresponds to a coumarin unit directly connected through an ester group to the polymeric backbone, whereas the second member integrates an ethandiyl linker. The SHG studies led on the first pair reveals two main features: the bit “0” level of **P13** is much lower than for **P9** and the contrast between bit “1” and bit “0” is larger. In the case of the second pair, a very significant contrast (ie gap between bit “1” and bit “0”) and a high SHG value are found in the case of the **P18** film, while those values are very low for **P3**. From these comparisons, it appears obvious that the addition of the ethandiyl linker results in a much better contrast between the SHG levels of the two bits, thus offering higher optical recording efficiency. This observation is ascribed to a better conformational mobility in the case of **P18** and **P13**, as promoted by the alkyl linker, which presumably leads to a higher efficiency of the intermolecular cyclodimerization reaction upon irradiation.

**Table 3:** SHG values for bit 1 and bit 0 and contrast for different coumarin-based polymers.

Polymer	SHG bit 1 (arb. units)	SHG bit 0 (arb. units)	Normalized SHG bit 1	Normalized SHG bit 0	Contrast (%)
**P9**	3.38	3.44	0.983	1	0.88
**P13**	3.23	3.34	0.967	1	1.67
**P3**	3.24	3.33	0.973	1	1.37
**P18**	4.2	8.75	0.480	1	35.14
**P2**	3.23	3.35	0.964	1	1.82
**P8**	2.33	2.41	0.967	1	1.69

A comparative study was also carried out between **P8** and **P2**, in order to address the effect of the NLO-active coumarin structure on the SHG contrast. Both copolymers are similar in terms of monomer ratio (n/m = 1/1) and do not present any linker between the polymeric backbone and the NLO-phore. They only differ by the structure of the latter, which is coumarin (**P2**) and methylcoumarin (**P8**) respectively. From these studies, it appears that coumarin derivative (**P2**) exhibits higher SHG values. Nevertheless, both materials **P2** and **P8** present similar and low contrasts, which indicates a very moderate influence of this structural parameter. An ultimate parameter concerns the impact of the (n/m) value. As mentioned above, the latter has a clear direct effect on the Tg value of the corresponding copolymers, which regularly decreases upon dilution of the coumarin units with increasing the ratio of MMA units (Table 1 and Table 2). As for the NLO contrast, no clear tendency could be drawn by comparing couples which only differ by the (n/m) ratio (*i.e.*, **P8** and **P9** on one hand or **P2** and **P3** on the other hand). Altogether, those comparative studies demonstrate the key importance of the conformational flexibility around the NLO-phore when designing the material. In other words, having a high SHG state for a given material is not enough for OSD purposes and a good contrast between both bits is required. This can be obtained through incorporation of a linker between the NLO-active unit and the polymer skeleton, which allows a more efficient dimerization reaction upon irradiation. Conversely, as mentioned above, one must keep in mind that introduction of a long alkyl chain results in low T_g_ values as in the case of **P14**, **P15**, **P17**, **P20**, **P21** (T_g_ = 40–80 °C) which integrate a hexandiyl linker, which are unsuitable for a stable SHG response. Finally, it appears obvious from these studies that the molecular system **P18** gets the closest behaviour to the ideal system and is the most promising candidate for binary ODS storage.

### 2.5. Image Storage

**Figure 2:**
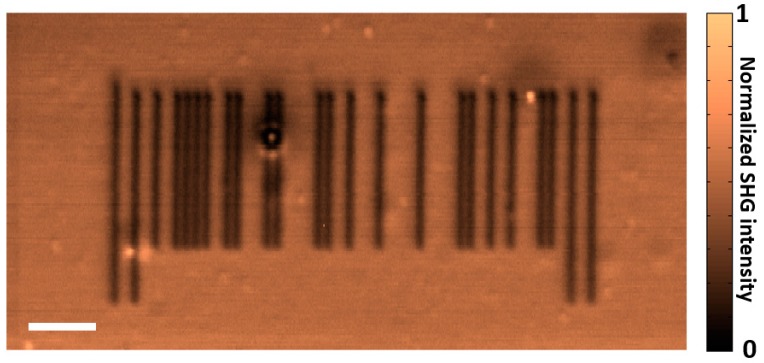
Normalized SHG image of a barcode (scale bar: 10 µm).

In order to demonstrate the possibility to perform ODS from a thin **P18** film, we recorded a 2D binary image (barcode, shown in Figure 2). During the recording process, the laser beam was switched on or off in each pixel accordingly to the value (black or white) of the pixel of the reference image (wavelength 700 nm, laser power 80 mW, writing scan rate 1 µm/s). The reading process of the stored binary information was done by SHG imaging (wavelength 800 nm, laser power 40 mW, scan rate 5 µm/s). The well-contrasted resulting SHG image corresponds to a film area of 100 × 50 μm². The dark areas correspond to dimerized-coumarin areas for which a lower SHG response is observed (bit “1”), while the brighter areas corresponds to non-dimerized ones (bit “0”) where the laser was switched off. Other ODS applications can be seen in previously published papers [22,24].

## 3. Experimental Section 

### 3.1. Characterization Techniques

Compounds **1a** and **2a** are commercially available. ^1^H-NMR (500 MHz) spectra were measured on a Bruker Avance DRX-500 spectrometer (Bruker Corp., Karlsruhe, Germany) using CDCl_3_ as solvent. Chemical shifts are given in ppm from the internal standard tetramethylsilane (TMS). The molecular weights of all polymers were obtained by Gel Permeation Chromatography with a SYSTEM AS1000 autosampler (Thermo Scientific Corp., Waltham, MA, USA), equipped with a guard column (PL gel 5 μm guard, 5 × 0.75 cm, Polymer Laboratories, Santa Clara, CA, USA) followed by 2 columns (Polymer Laboratories, 2 PL gel 5 μm MIXED-D columns, 0.2 × 30 × 0.75 mm), with a SpectraSYSTEM RI-150 and a SpectraSYSTEM UV2000 detectors. The eluent used is THF at a flow rate of 1 mL·min^−1^ at 35 °C. Polystyrene standards (580–4.83 g·mol^−1^) were used for calibration. The mass spectra were obtained on a Trace DSQ GC-MS mass spectrometer (Thermo Scientific Corp.). The glass transition temperatures (T_g_) of all polymers are determined by Differential Scanning Calorimetry with Q20 Differential Scanning Calorimetry model (TA Instruments, New Castle, PA, USA) with a continuous N2 purge. The sample was initially stabilized and after the first scan was made at a heating rate of 10 °C/min up to 200 °C then cooled to 20 °C, a second scan was performed with the same parameters to obtain the values of Tg. UV-Vis spectroscopy measurements were performed at room temperature with thin films deposited on glass substrates with a UV/Vis/NIR Lambda 19 spectrophotometer (PerkinElmer Corp., Waltham, MA, USA). For irradiation experiments, the thin films were irradiated at 20 °C with a UV immersion lamp TQ 150 W (Heraeus, Hanau, Germany) equipped with a filter (Duran 50, Heraeus) that cuts off wavelengths below 300 nm. The photocleavage of coumarin dimers was achieved using a low pressure mercury lamp TNN 15/32 (Heraeus), 15 W with emission wavelength spectrum at 254 nm (20 °C, 30 min). In both cases, a 10 cm distance was kept between the lamp and the surface of film during the irradiation.

### 3.2. Thin Films Preparation

The spin-coating deposition technique was used to produce thin films of coumarin-based copolymers **P1**–**P21** with a controlled thickness, on glass slides BK7. Polymer films were spin-coated at 800 rpm from 10 wt % 1,1,2-trichloroethane solution previously filtered through a 400 nm pore size nylon syringe filter. Immediately after deposition, the thin films were dried in vacuum at 35 °C for 5 h to eliminate any remaining solvent. The thickness of the thin solid films was measured by a profilometer (Dektak 6M Stylus Surface Profilometer, Veeco, Plainview, NY, USA) and was found to be about 0.8–1.2 μm for all samples.

### 3.3. Monomer Synthesis

#### 3.3.1. *7-(2-Hydroxyethoxy)coumarin* (**1b**)

A solution of 2-bromoethanol (127 mmol), 7-hydroxycoumarin (**1a**, 10.54 g, 65 mmol) and potassium carbonate (50.44 g, 365 mmol) in acetone (250 mL) was heated at 80 °C for 65 h. The cooled reaction solution was filtered and then evaporated under vacuum. The crude product was dissolved in dichloromethane and treated with a saturated ammonium chloride solution. The aqueous phase was extracted with dichloromethane and the combined organic phases where washed with water, dried over MgSO_4_ and the solvent was evaporated under vacuum. The crude material was then purified by column chromatography on silica gel (ethyl acetate) to afford a white powder after solvent evaporation; m.p. 89–90 °C, yield 51%. ^1^H-NMR (500 MHz, CDCl_3_) δ (ppm): 1.98 (t, 1H, OH), 4,02 (m, 2H, -CH_2_OH), 4.15 (t, 2H, -CH_2_-OAr), 6.27 (d, 1H, *J* = 9.5 Hz, enone), 6.84–6.88 (m, 2H, ArH), 7.39 (d, 1H, Ar-H), 7.64 (d, 1H, *J* = 9.5 Hz, enone). [22]

#### 3.3.2. *7-[(6-Hydroxyhexyl)oxy]coumarin* (**1c**)

6-Bromohexan-1-ol (1.57 ml; 12 mmol) was added to a mixture of 7-hydroxycoumarin (**1a**, 12 mmol), anhydrous potassium carbonate (5.5 g; 40 mmol) and a catalytic amount of potassium iodide in a round-bottomed flask with freshly distilled acetone (75 mL). The mixture was heated at reflux for 30 h. The solid was filtered off and the solvent was evaporated to dryness under vacuum. The crude product was purified by column chromatography on silica gel (hexane–ethyl acetate, 4:6) to afford **1c** as an oil after solvent evaporation; yield 89%. ^1^H-NMR (500 MHz, CDCl_3_): δ 1.2–1.95 (m, 8H, -CH_2_CH_2_CH_2_CH_2_-), 3.7 (t, 2H, -CH_2_OH), 4.00 (t, 2H, -CH_2_-OAr), 6.2 (d, 1H, =CH-C(O)-), 6.81 (m, 2H, Ar-H), 7.4 (d, 1H, Ar-H), 7.6 (d, 1H, Ar-H).

#### 3.3.3. *7-(2-Hydroxyethoxy)4-methylcoumarin* (**2b**)

A solution of 2-bromoethanol (1.36 mL, 19 mmol), 7-hydroxy-4-methylcoumarin (**2a**, 2.33 g, 13 mmol), potassium carbonate (8.32 g, 60 mmol), and acetone (50 mL) was heated at 80 °C for 30 h. The cooled reaction solution was filtered and then evaporated under vacuum. The crude product was purified using column chromatography on silica gel with ethyl acetate as eluent; yield 91%. ^1^H-NMR (500 MHz, CDCl_3_): δ 1.27 (t, 1H, OH), 2.41 (s, 3H, -CH_3_), 4.03 (m, 2H, -CH_2_OH), 4.16 (t, 2H, -CH_2_-OAr), 6.15 (d, 1H, =CH-C(O)-), 6.83–6.90 (m, 2H, ArH), 7.50 (d, 1H, Ar-H). 

#### 3.3.4. *7-[(6-Hydroxyhexyl)oxy]-4-methylcoumarin* (**2c**)

The same procedure as for **1c** was used, but employing 7-hydroxy-4-methylcoumarin (**2a**). The crude product was purified by column chromatography on silica gel (hexane–ethyl acetate, 4:6), to afford **2c** as white crystals; m.p. 81–82 °C, yield 82%. ^1^H-NMR (500 MHz, CDCl_3_): δ 1.40–1.85 (m, 8H, -CH_2_CH_2_CH_2_CH_2_-), 2.38 (s, 3H, -CH_3_), 3.66 (t, 2H, -CH_2_OH), 4.00 (t, 2H, -CH_2_-OAr), 6.17 (d, 1H, =CH-C(O)-), 6.81 (t, 2H, Ar-H), 7.48 (d, 1H, Ar-H). MS, *m*/*z*: 276 [M]^+^.

#### 3.3.5. *7-Methacryloyloxycoumarin* (**3a**) 

7-Hydroxycoumarin (**1a**, 17 mmol) was dissolved in *N*,*N*-dimethylformamide (DMF, 20 mL) and triethylamine (20 mmol) was added. The reaction mixture was cooled to 0–5 °C and methacryloyl chloride (1.98 mL; 20 mmol) in DMF (5 mL) was injected gradually to the above solution. After 4 h at reflux, the solution was poured on ice. The resulting solid product was filtered off, washed with water and dried. Recrystallization from ethanol gave colorless plates; m.p. 146–147 °C, yield 80%. ^1^H-NMR (500 MHz, CDCl_3_): δ 2.0 (s, 3H, -CH_3_), 5.9 (s, 1H, CH_2_=), 6.5 (d, 1H, =CH-C(O)-), 6.35 (s, 1H, CH_2_=), 7.2 (d, 1H, Ar-H), 7.3 (s, 1H, Ar-H), 8.1 (d, 1H, Ar-H), 7.8 (d, 1H, Ar-H). 

#### 3.3.6. *7-(2-Methacryloyloxyethoxy)coumarin* (**3b**)

This compound was prepared according to [22].

#### 3.3.7. *7-[6-Methacryloyloxyhexyloxy]coumarin* (**3c**)

7-[(6-Hydroxyhexyl)oxy]coumarin (**1c**, 1.33 g; 4.8 mmol) was dissolved in dry methylene chloride (30 mL). The reaction mixture was kept at 0–5 °C and triethylamine (0.68 mL; 5 mmol) and methacryloyl chloride (0.48 mL; 5 mmol) were added gradually. The solution was refluxed for 4 h, washed (1 M hydrochloric acid, 10% sodium hydroxide) and then dried over anhydrous magnesium sulfate. The crude oil was purified by column chromatography on silica gel using methylene chloride as eluent; yield 50%. ^1^H-NMR (500 MHz, CDCl_3_): δ 1.2–1.9 (m, 8H, -CH_2_CH_2_CH_2_CH_2_-), 2.0 (s, 3H, H_2_C=C(CH_3_)-), 4.00 (t, 2H, -CH_2_-OPh), 4.2 (t, 2H, -CH_2_-O-C(O)-), 5.6 (s, 1H, CH_2_=), 6.1 (s, 1H, CH_2_=), 6.2 (d, 1H, =CH-C(O)-), 6.80–6.83 (m, 2H, Ar-H), 7.4 (d, 1H, Ar-H), 7.6 (d, 1H, Ar-H).

#### 3.3.8. *7-Methacryloyloxy-4-methylcoumarin* (**4a**)

Same procedure as for **3a** was employed, but using 7-hydroxy-4-methylcoumarin (**2a**). Recrystallization from ethanol gave colorless plates m.p. 177 °C, 85%. ^1^H-NMR (500 MHz, CDCl_3_): δ 2.05 (s, 3H, -CH_3_), 2.41 (s, 3H, -CH_3_), 5.80 (s, 1H, CH_2_=), 6.23 (d, 1H, =CH-C(O)-), 6.35 (s, 1H, CH_2_=), 7.09 (t, 2H, Ar-H), 7.60 (d, 1H, Ar-H). MS, *m*/*z*: 244 [M]^+^.

#### 3.3.9. *7-(2-Methacryloyloxyethoxy)4-methylcoumarin* (**4b**)

Methacryloyl chloride (0.48 mL) was added dropwise to a mixture of triethylamine (0.69 mL) and 7-(2-hydroxyethoxy)4-methylcoumarin (**2b**) in THF (40 mL). After stirring for 12 h at 0 °C, the reaction mixture was diluted with dichloromethane and the organic layer was washed with an aqueous solution of NaCl and dried over magnesium sulfate, to produce a solid after solvent evaporation. The crude product was recrystallized from ethanol to afford a colorless microcrystalline solid; 80% yield. ^1^H-NMR (500 MHz, CDCl_3_): δ 1.96 (s, 3H, CH_3_), 2.4 (s, 3H, CH_3_), 4.28 (t, 2H, CH_2_OR), 4.53 (t, 2H, CH_2_OAr), 5.60 (s, 1H, ethylene), 6.15 (s, 1H, ethylene; d, 1H, *J*), 6.8–6.9 (m, 2H, Ar-H), 7.5 (d, 1H, Ar-H).

#### 3.3.10. *7-[[[6-(Methacryloyl)oxy]hexyl]oxy]-4-methylcoumarin* (**4c**)

This compound was prepared according to [23].

### 3.4. Polymerization

The copolymers **P1**–**P21** were synthesized by free radical polymerization using coumarin methacrylic monomers and methyl methacrylate (MMA) or butyl methacrylate (BMA) in 10% DMF solution with AIBN as radical initiator at 80 °C (argon atmosphere). The mixture was degassed with repeated freeze and thaw cycles and then heated for 24 h. The resulting viscous solution was added into methanol to precipitate a polymeric material. The precipitation was repeated from DMF into methanol to give the purified polymer. The copolymerization ratio was calculated on the basis of the integration ratio of ^1^H-NMR measurements (Table 1 and Table 2).

### 3.5. Molecular Orientation in Thin Films by Corona Poling

**Figure 3:**
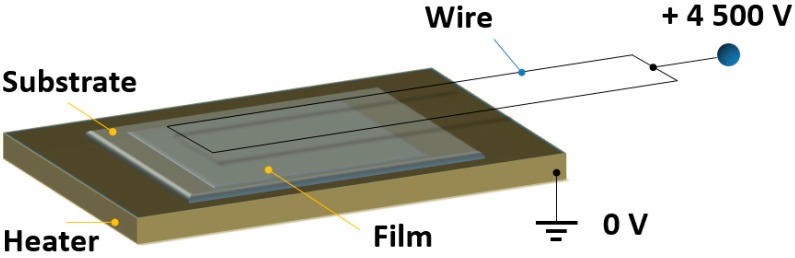
Corona-poling setup. The substrate is a BK7 glass slide. The distance between the wire and the film is approximately 1 cm.

The dipole moments of the chromophores are initially randomly oriented within the film and as a result, the macroscopic second order nonlinear optical response of the material is negligible. To overcome this problem the molecules are oriented by the corona-poling technique [26,28] as illustrated in Figure 3. The film is heated up to a temperature slightly lower than the glass transition temperature of the polymer and a strong electric field (4.5 kV) is applied thanks to two 30 μm tungsten wires which are placed over the sample. The sample is kept under these conditions for ten minutes. While keeping the electric field constant, the temperature is smoothly decreased down to room temperature, resulting to the orientation of the dipole moments and inducing a non-centrosymmetry on the sample, required for a second order nonlinear optical response. The uniformly poled film can be considered as a “blank”, exhibiting homogeneous maximum SHG, so all the bits over the surface are set to “0”. Due to the relatively high glass transition temperatures of the films, the orientation of the molecules (and SHG values) are expected to be maintained even several months after the corona poling (6 months, as checked in the case of **P18**).

### 3.6. Non-Linear Microscopy Setup

**Figure 4:**
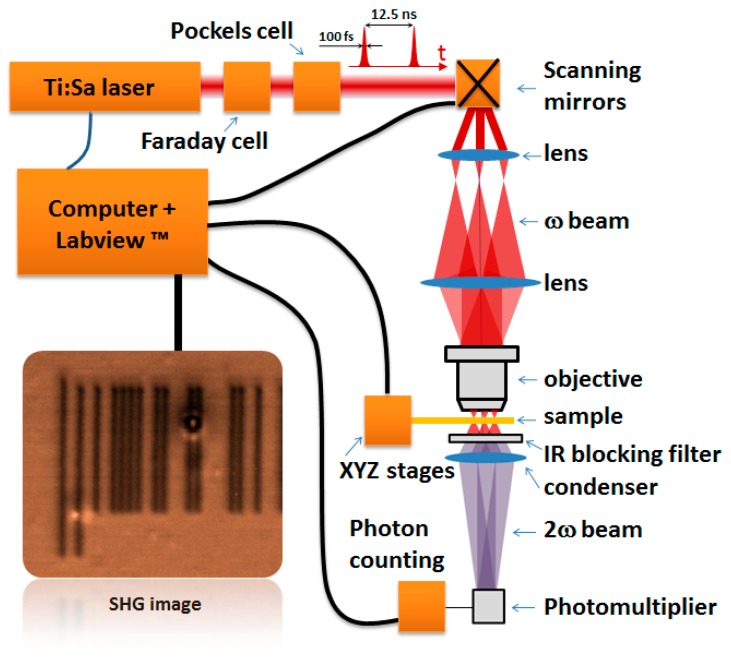
Non-linear scanning microscopy setup used to induce localized photo-dimerization and to detect the SHG response.

A schematic representation of the setup is shown in Figure 4. The writing process (cyclodimerization induced by two-photon absorption) and read-out process (SHG imaging) are performed using a tunable (670–1100 nm) Ti:sapphire laser source (Spectra-Physics Tsunami, 120 fs pulse duration, 80 MHz repetition rate, linearly polarized) pumped by a 10 W solid-state laser (Millenia Xs, Spectra Physics). The laser power was adjusted by means of an electro-optic modulator (Pockels cell, Conoptics, Danbury, CT, USA). A Faraday rotator was used as an optical isolator. The angular deviation of the infrared beam was controlled with an X-Y scanner (Cambridge Technology, Bedford, MA, USA) and then focused on the sample by a long working distance microscope objective (Olympus SLMPLN20X, numerical aperture = 0.25). The lateral resolution is about 1 µm for the illuminated area. The transmitted NLO signal (SHG) is filtered with IR blocking filters (NT48-071, Edmund Optics Ltd., York, UK, and BG39, Schott, Mainz, Germany) and detected by a fast photomultiplier (H744P-40, Hamamatsu) coupled with a photon counter (C9744, Hamamatsu, Hirakuchi, Japan). The samples (thin films deposited on glass microscope slides) are placed on X, Y, Z, motorized stages (Newport, Irvine, CA, USA) and driven by a high performance 8-axes motion controller (XPS, Newport) and computer controlled in order to keep always the focal point in the plane of the sample [24]. We have developed a Labview™ program (National Instrument, Austin, TX, USA) to control the micro-positioning of the sample, the light polarization, the electric signals for the scanner and the photon counter in order to store the signal for each position of the scanning laser beam on the sample and to produce an SHG image, pixel after pixel, at the maximum frequency of 1 MHz.

## 4. Conclusions

A comparative study of several coumarin-based copolymers was performed in terms of their efficiency for optical data storage applications. The ODS was carried out by locally modifying the SHG response of these materials by means of two-photon absorption, while the efficiency of each film was related with the SHG contrast between bits “0” and “1”. A relation between the molecular structure and the ODS efficiency has been established and demonstrates the critical importance of the molecular design. In particular, introduction of a short linker between the polymer backbone and the pendant coumarin unit allows a better conformational freedom of the latter, which results in better contrasts between SHG of the monomeric and the dimeric forms. This improvement is assigned to a higher efficiency of the cyclodimerization reaction.

## Supplementary Materials

Supplementary materials can be accessed at: http://www.mdpi.com/1420-3049/21/2/147/s1.
