# Supplementary material for: Coumarin-Containing Polymers for High Density Non-Linear Optical Data Storage
Source: Molecules. 2016 Jan 26;21(2):147. doi: 10.3390/molecules21020147 (PMC6273180; doi:10.3390/molecules21020147)
Supplement: Supplementary file 1 [file molecules-21-00147-s001.pdf]

Supplementary Materials: Coumarin-Containing Polymers for High Density Non Linear Optical Data Storage

Denis Gindre, Konstantinos Iliopoulos, Oksana Krupka, Marie Evrard, Emilie Champigny and Marc Sallé

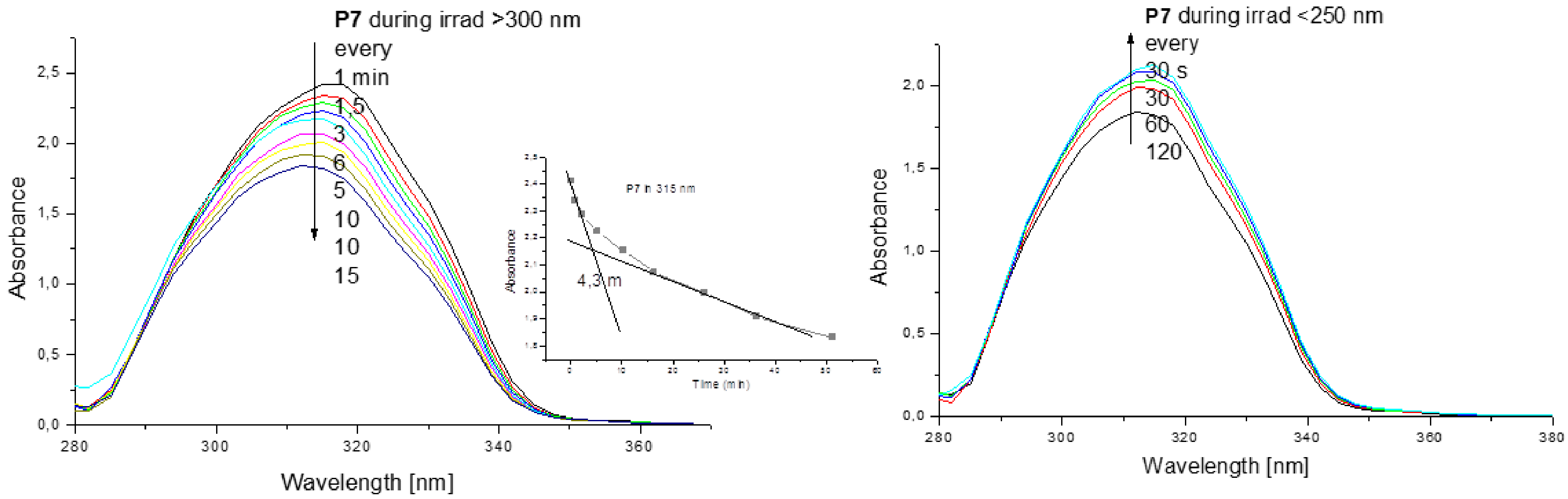

Figure S1. *Cont.*

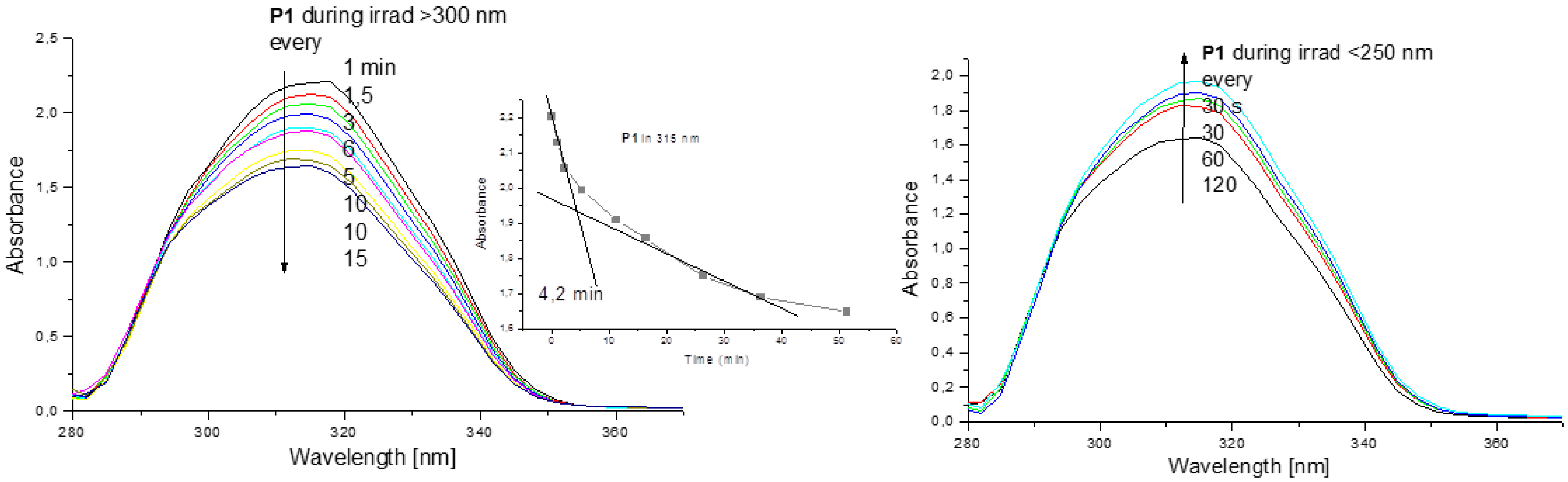

Figure S1. Changes in the absorption spectrum of polymer **P7** and **P1** in thin film before (1) and after periods of irradiation at $\lambda > 300$ nm; Changes in the absorption of film polymers at maximum of absorption during irradiation at $\lambda > 300$ nm; Changes of the absorption spectrum cross-linked polymer in thin solid film before (1) and after time of UV irradiation at $\lambda < 254$ nm.

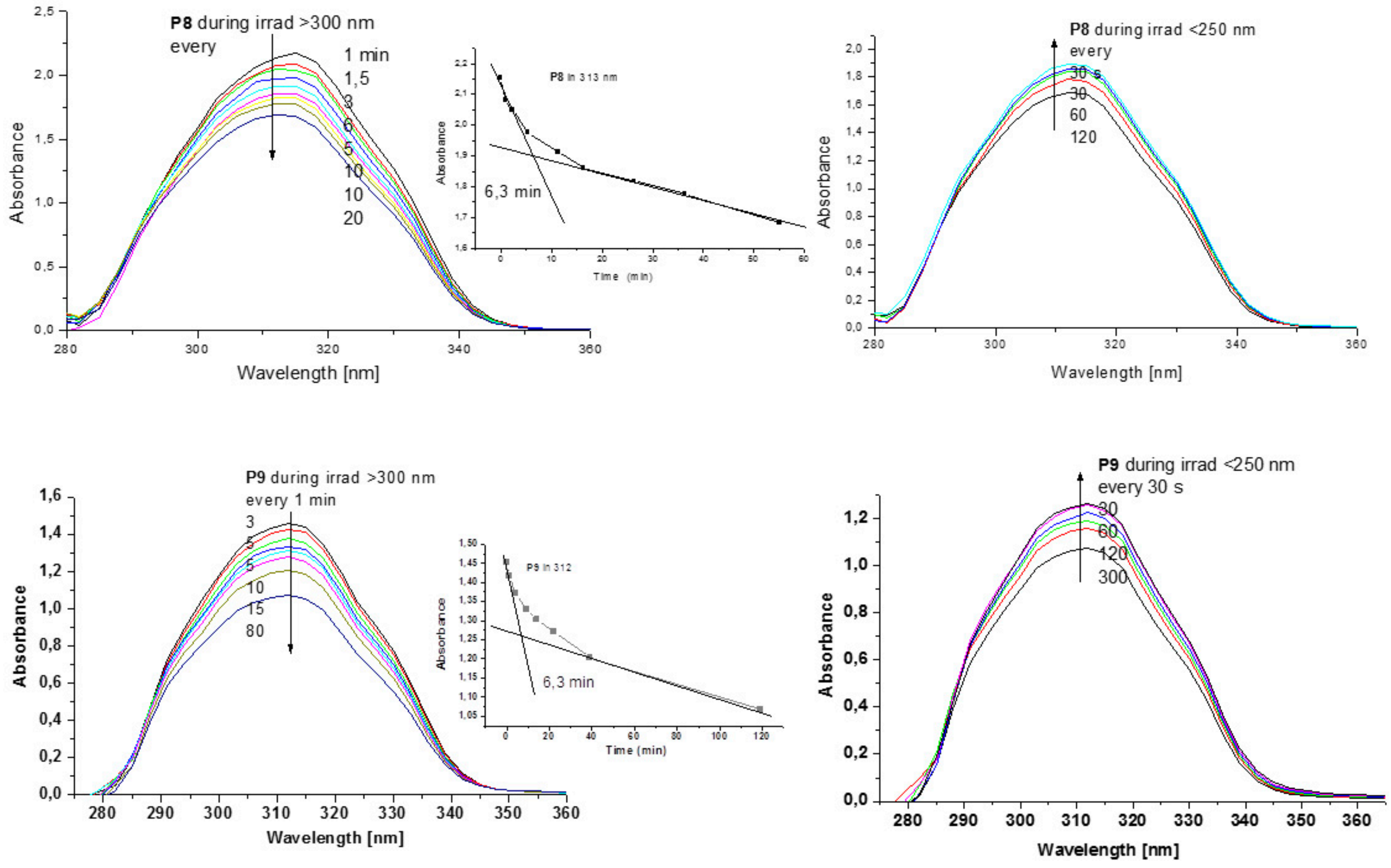

Figure S2. Changes in the absorption spectrum of polymer **P8** and **P9** in thin film before (1) and after periods of irradiation at $\lambda > 300$ nm; Changes in the absorption of film polymers at maximum of absorption during irradiation at $\lambda > 300$ nm; Changes of the absorption spectrum cross-linked polymer in thin solid film before (1) and after time of UV irradiation at $\lambda < 254$ nm.

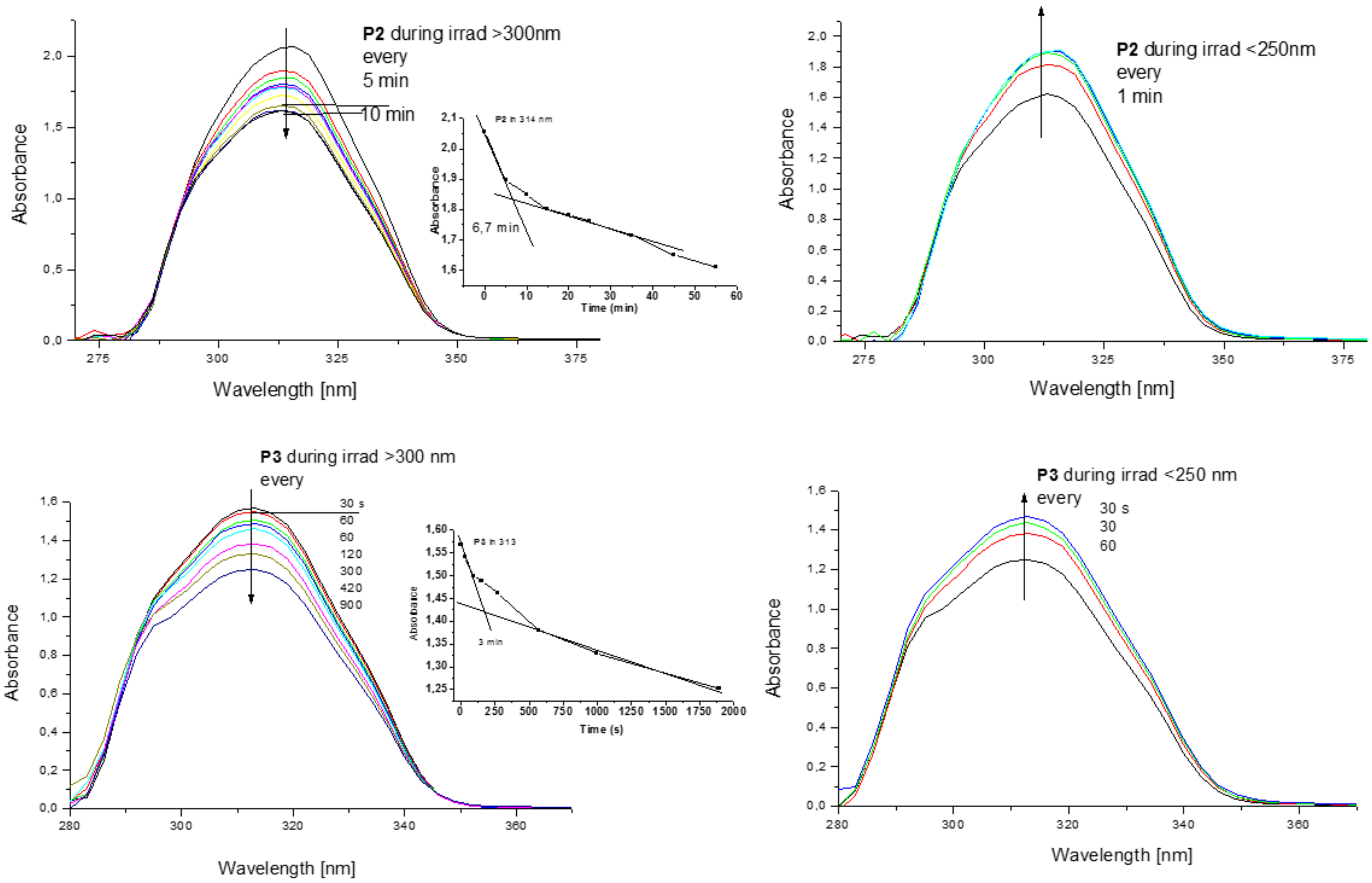

Figure S3. Changes in the absorption spectrum of polymer **P2** and **P3** in thin film before (1) and after periods of irradiation at $\lambda > 300$ nm; Changes in the absorption of film polymers at maximum of absorption during irradiation at $\lambda > 300$ nm; Changes of the absorption spectrum cross-linked polymer in thin solid film before (1) and after time of UV irradiation at $\lambda < 254$ nm.

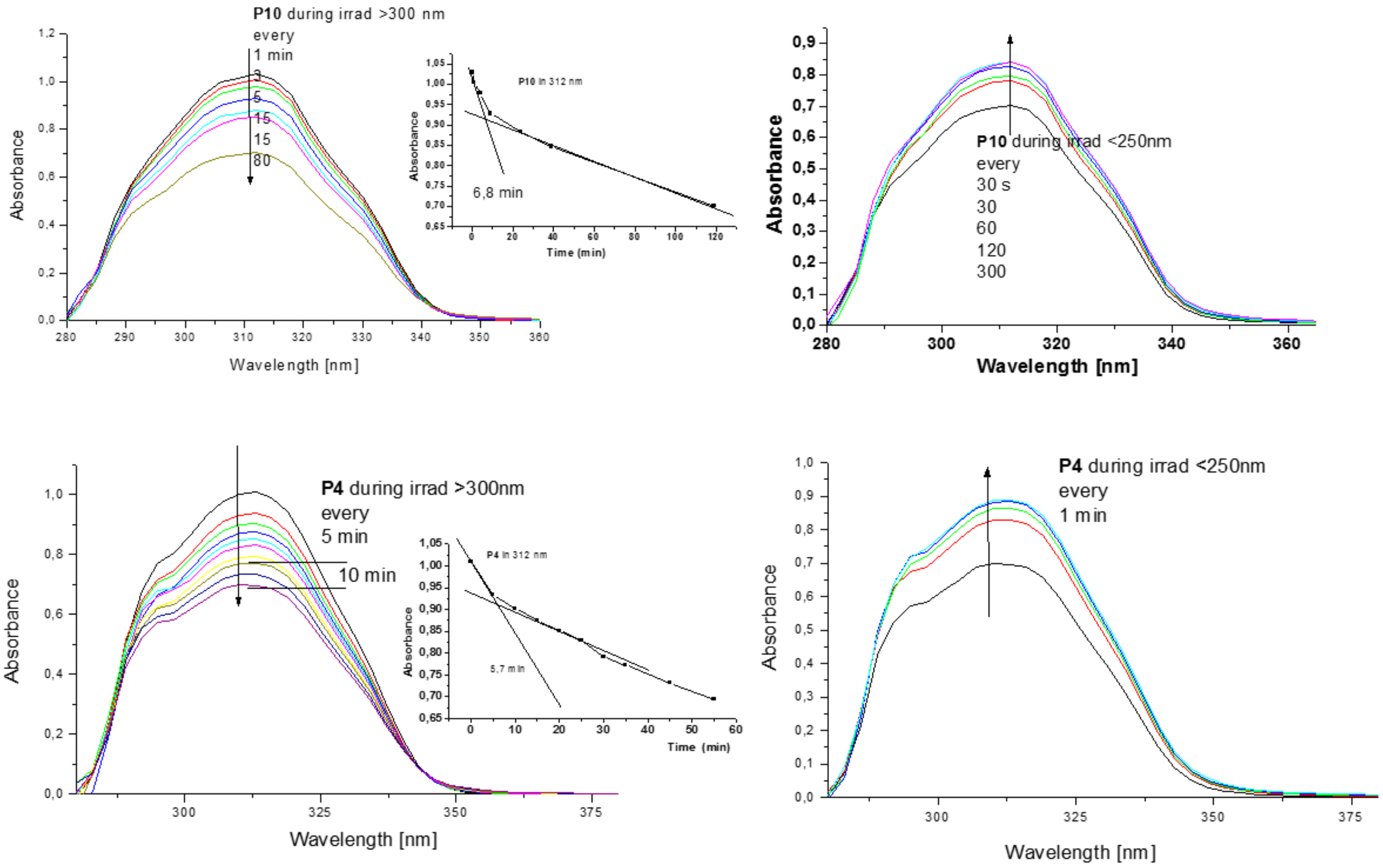

Figure S4. Changes in the absorption spectrum of polymer **P10** and **P4** in thin film before (1) and after periods of irradiation at $\lambda > 300$ nm; Changes in the absorption of film polymers at maximum of absorption during irradiation at $\lambda > 300$ nm; Changes of the absorption spectrum cross-linked polymer in thin solid film before (1) and after time of UV irradiation at $\lambda < 254$ nm.

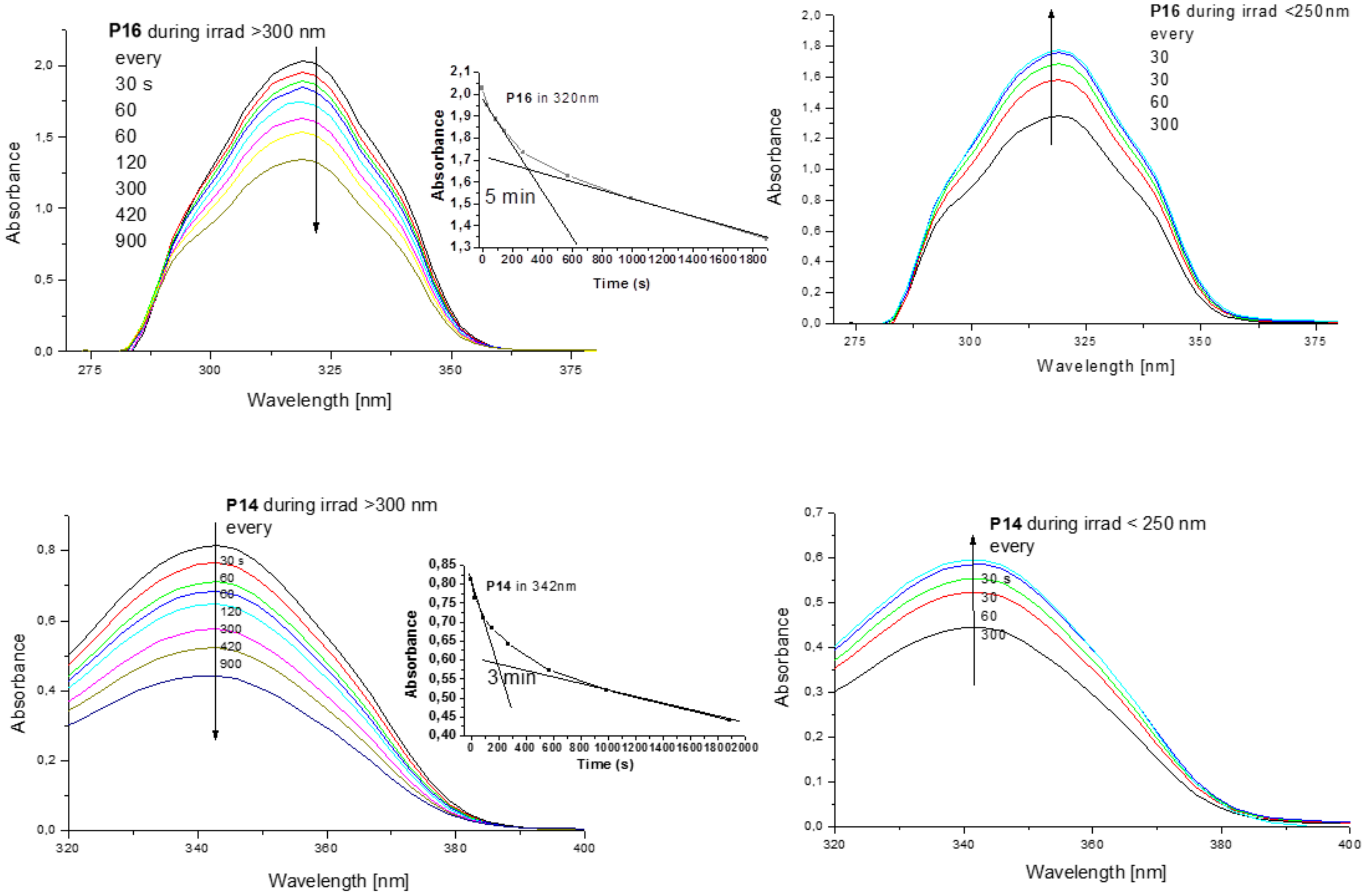

Figure S5. Changes in the absorption spectrum of polymer **P16** and **P14** in thin film before (1) and after periods of irradiation at $\lambda > 300$ nm; Changes in the absorption of film polymers at maximum of absorption during irradiation at $\lambda > 300$ nm; Changes of the absorption spectrum cross-linked polymer in thin solid film before (1) and after time of UV irradiation at $\lambda < 254$ nm.

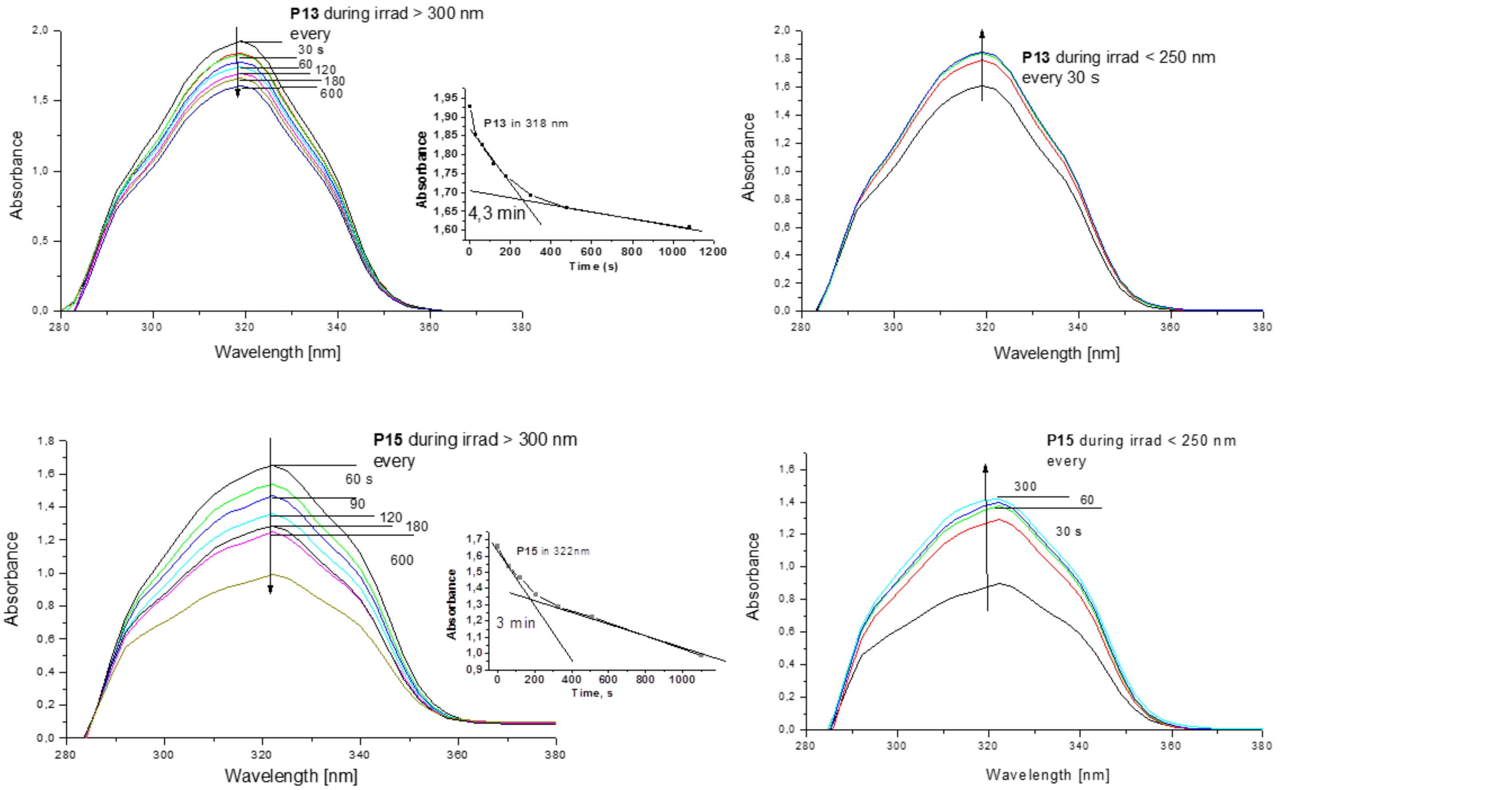

Figure S6. Changes in the absorption spectrum of polymer **P13** and **P15** in thin film before (1) and after periods of irradiation at $\lambda > 300$ nm; Changes in the absorption of film polymers at maximum of absorption during irradiation at $\lambda > 300$ nm; Changes of the absorption spectrum cross-linked polymer in thin solid film before (1) and after time of UV irradiation at $\lambda < 254$ nm.
